# Comparative Success of Recruitment Strategies for an Exercise Intervention Trial Among Women With Polycystic Ovary Syndrome: Observational Study

**DOI:** 10.2196/25208

**Published:** 2021-03-30

**Authors:** Jamie L Benham, Jane E Booth, Christine M Friedenreich, Doreen M Rabi, Ronald J Sigal

**Affiliations:** 1 Department of Medicine Cumming School of Medicine University of Calgary Calgary, AB Canada; 2 Department of Community Health Sciences Cumming School of Medicine University of Calgary Calgary, AB Canada; 3 Department of Cancer Epidemiology and Prevention Research Cancer Care Alberta Alberta Health Services Calgary, AB Canada; 4 Department of Oncology Cumming School of Medicine University of Calgary Calgary, AB Canada; 5 Faculty of Kinesiology University of Calgary Calgary, AB Canada; 6 Department of Cardiac Sciences Cumming School of Medicine University of Calgary Calgary, AB Canada

**Keywords:** aerobic exercise, exercise, exercise training, ovary, polycystic ovary syndrome, recruitment, well-being, women’s health

## Abstract

**Background:**

Effective and efficient participant recruitment is a key determinant of the success of a research program. Previously reported recruitment strategies have displayed variable success rates in studies on women with polycystic ovary syndrome (PCOS).

**Objective:**

This study aimed to evaluate the effectiveness and cost per participant of the recruitment strategies that we used in a prospective randomized controlled trial to examine the effects of exercise training among inactive women with PCOS, who are aged 18-40 years.

**Methods:**

The 4 recruitment methods we used were as follows: (1) referral by health care providers or by word of mouth, (2) media (eg, local newspaper stories and radio interviews), (3) Facebook advertisements, and (4) unpaid advertisements including posters and websites. The proportions of potential, eligible, and enrolled participants recruited with each method were determined and compared using tests of proportion. The time investment and cost per participant enrolled were calculated for each recruitment strategy.

**Results:**

Of 200 potential participants screened, 98 (49%) were recruited from unpaid advertisements (posters and websites), 70 (35%) from Facebook advertisements, 16 (8%) by referral, and 16 (8%) from traditional media (newspaper and radio). Every potential participant was recruited from separate means (ie, no participant was approached through more than one recruitment method). A total of 109 (54.5%) women were deemed eligible for participation in the trial, and 60 (30.0%) were enrolled. The proportion of potential participants who completed the trial was higher for those recruited from traditional media than from Facebook advertisements (n=7/16, 44% vs n=13/70, 19%, respectively; *P*=.03) or unpaid advertisements (n=7/16, 44% vs n=13/98, 13%, respectively; *P*=.002). The cost per participant was Can $18.21 (US $14.46) for Facebook advertisements and Can $43.88 (US $34.85) for unpaid advertisements. There were no direct trial costs for referrals or traditional media.

**Conclusions:**

For this trial, each method was important for recruiting inactive women with PCOS because no participant reported learning about the trial through more than one method. Unpaid advertisements and Facebook advertisements helped recruit the largest number of participants in the trial, the former resulting in a higher cost per participant than the latter.

**Trial Registration:**

ClinicalTrials.gov NCT03362918; https://clinicaltrials.gov/ct2/show/NCT03362918

## Introduction

Polycystic ovary syndrome (PCOS) is a common endocrine disorder that affects up to 1 in 5 women of reproductive age [1]. Lifestyle interventions including exercise training and dietary modifications are encouraged for the management of PCOS [2], yet the optimal exercise prescription is unknown [3]. To address this issue, we designed a randomized controlled trial to evaluate the effect of 2 forms of exercise training—high-intensity interval training (HIIT) and continuous aerobic exercise training (CAET)—on the reproductive, anthropometric, and cardiometabolic outcomes of women of reproductive age living with PCOS compared to those in a no-exercise control group.

The ability to recruit participants effectively while simultaneously meeting the inclusion and exclusion criteria of a research program in a timely manner is a key determinant of the trial’s ultimate success. Previous trials among women with PCOS employed various strategies to recruit participants, including referrals by health care providers, web-based advertisements, posters, and Facebook advertisements, all of which have displayed variable effectiveness [4-6]. Qualitative studies have reported that advertising on social media platforms and posting flyers at gynecology clinics might be effective strategies to recruit women living with PCOS to participate in a research study [7]. While advertising on social media platforms has been identified as a potentially effective recruitment strategy, the relative cost per participant from using Facebook advertisements to recruit women with PCOS has not been compared with that of more traditional strategies such as physician referrals and paid advertisements.

This study aimed to compare the success of 4 strategies used to recruit physically inactive women of reproductive age living with PCOS to a behavioral intervention trial.

## Methods

### Study Design

We conducted a single-center prospective randomized controlled trial to assess the effect of 2 forms of exercise training—high-intensity interval training (HIIT) and continuous aerobic exercise training (CAET)—on the reproductive, anthropometric, and cardiometabolic health markers in women with PCOS compared to those in a no-exercise control group [8]. The study period was 15 months and comprised 3 phases: (1) a 3-month run-in phase to assess baseline reproductive function; (2) a 6-month intervention phase where participants were randomized into HIIT, CAET, or no-exercise control groups; and (3) a 6-month follow-up phase. The run-in and intervention phases required daily assessment of menstrual status and ovulation. During the intervention phase, the participants randomly assigned to exercise training were prescribed 3 exercise sessions per week. The Conjoint Health Research Ethics Board of the University of Calgary and Alberta Health Services (REB17-1574) approved the study, and all participants provided written informed consent.

### Participants

Physically inactive women with PCOS between the ages of 18 and 40 years were recruited in this study. The exclusion criteria were as follows: (1) taking medication that interferes with ovulation (eg, estrogens, progestins, glucocorticoids, metformin, gonadotropins, clomiphene citrate, or letrozole), (2) participating in regular exercise training for >40 minutes per week, and (3) having medical conditions that prevent them from receiving exercise training. Potential participants were initially screened through a telephone call on the basis of the inclusion and exclusion criteria and then through screening investigations including blood tests and electrocardiography. On meeting all trial inclusion and exclusion criteria, potential participants were invited for an in-person assessment and enrolled in the trial.

### Recruitment Strategies

Recruitment for the trial was carried out between December 2017 and December 2018 in Calgary (Alberta, Canada) through 4 recruitment strategies simultaneously: Facebook advertisements, unpaid advertisements, traditional media, and referrals.

#### Facebook Advertisements

**Figure 1:**
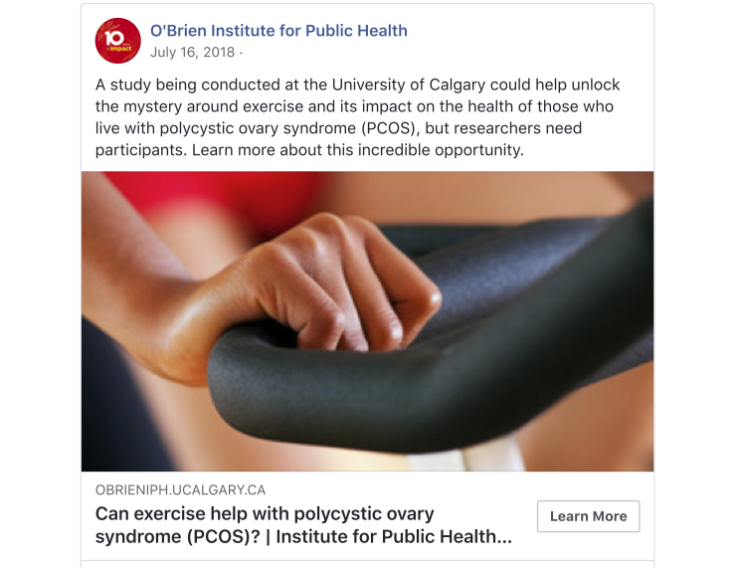
Facebook advertisement run in July 2018.

In total, 3 paid Facebook advertisements were used during trial recruitment. Each advertisement featured a cropped image of an individual performing exercise training and was captioned with the following phrase: “Can exercise help with Polycystic Ovary Syndrome (PCOS)?” with a click-through to a landing page that featured details on trial eligibility. The advertisements were consistent with Facebook’s advertising policy [9]. The advertisements were targeted in accordance with location (ie, Calgary) and demographics (ie, women aged 18-40 years). We did not target advertisements on the basis of interests, behaviors, or connections. The first 2 advertisements were run for 2 weeks each in April and July 2018 (Figure 1) and the final advertisement was run for 1 week in September 2018.

#### Unpaid Advertisements

We used printed posters and websites. The printed posters featured an image of several individuals cycling indoors, and the title read: “Do you have Polycystic Ovary Syndrome (PCOS)? Would you like to become more physically active?” They were displayed locally on public poster boards at various sites including coffee shops, grocery stores, university campuses, public libraries, and recreation centers. Information regarding the trial was also posted on the following websites: (1) the University of Calgary research services office website [10]; (2) the O’Brien Institute of Public Health, Cumming School of Medicine, University of Calgary website [11]; and (3) ClinicalTrials.gov [12].

#### Traditional Media

Several research team members involved in the trial were interviewed about the trial for a University of Calgary publication “UToday.” A member of the research team participated in broadcast interviews about the trial on 2 local radio stations.

#### Referrals

Local endocrinologists, gynecologists, family physicians, personal trainers, dieticians, and naturopaths were provided with information about the trial to disseminate to their patients who might meet the eligibility criteria of the trial. Participants were also recruited through word of mouth.

### Recruitment Costs

Over the course of recruitment for the trial, costs incurred directly by the trial for each recruitment strategy were recorded. The time that trial team members spent on each of the recruitment methods was approximated and multiplied by the hourly compensation rate of the research assistant (Can $25 [US $19.86]). The cost of poster printing was estimated.

### Statistical Analysis

The number of potential participants recruited through each recruitment method was determined. The proportions of potential participants who were eligible for enrollment, enrolled, randomized, and completed the trial was calculated for each recruitment method (Table 1). Tests of proportion were used to compare between-group differences. The time investment per enrolled participant was calculated by dividing the total number of hours spent on a recruitment strategy by the number of participants enrolled using that strategy. For each recruitment strategy, the cost per participant was determined by dividing the total cost of the recruitment strategy by the total number of participants enrolled using that strategy. Statistical significance was set at *P*<.05. The analysis was conducted using STATA (version 15.1, StataCorp).

**Table 1:** Outcome measure definitions.

Outcome measure	Definition
Participants recruited	The number of potential participants recruited to the trial.
Proportion of participants eligible for enrollment	The number of potential participants who were eligible to be enrolled in the trial divided by the total number of participants recruited.
Enrolled in the trial	The number of participants who were enrolled in the trial divided by the total number of participants recruited.
Randomized	The number of participants who completed the 3-month run-in phase and then randomized divided by the total number of participants recruited.
Completed the trial	The number of participants who completed the 9-month trial divided by the total number of participants recruited.
Time investment per participant	The total number of hours spent on a recruitment strategy divided by the number of participants enrolled using that strategy.
Cost per participant	The total cost of a recruitment strategy divided by the total number of participants enrolled using that strategy.

## Results

### Results Summary

Of the 200 potential participants screened for this pilot trial, 98 (49%) were recruited from unpaid advertisements, 70 (35%) from Facebook advertisements, 16 (8%) from traditional media, and 16 (8%) through referrals (Table 2). We observed no between-group differences in the proportion of participants who were eligible for enrollment or those who were enrolled in the trial. The proportion of potential participants who completed the 3-month run-in phase and then randomized was significantly greater for those recruited from traditional media than for those recruited from Facebook advertisements (n=7/16, 44% vs n=5/70, 24%, respectively; *P*=.03). The proportion of potential participants who completed the trial was significantly greater for those recruited from traditional media than for those recruited from Facebook advertisements (n=7/16, 44% vs n=13/70, 19%, respectively; *P*=.03) or unpaid advertisements (n=7/16, 44% vs n=13/98, 13%, respectively; *P*=.002). No participants reported learning about the trial from more than 1 recruitment method.

**Table 2:** Recruitment and completion of an exercise trial for women with polycystic ovary syndrome in Calgary (Alberta, Canada) in 2017-2020 (N=60).

Condition	All recruitment methods	Unpaid advertisements^a^	Facebook advertisements	Traditional media^b^	Referral^c^
Recruited, n	200	98	70	16	16
Eligible for enrollment, n (%)	109 (55.0)	49 (50)	39 (56)	8 (50)	10 (63)
Enrolled in the trial, n (%)	60 (30.0)	25 (26)	21 (30)	7 (44)	7 (44)
Randomized, n (%)	47 (24.0)	19 (19)	15 (21)	7 (44)	6 (38)
Completed the trial, n (%)	37 (19.0)	13 (13)	13 (19)	7 (44)	4 (25)
Age of the enrolled participants (years), mean (SD)	29.5 (4.9)	29.2 (5.2)	31.0 (4.4)	27.2 (5.5)	27.8 (3.1)
Employed, n (%)	54 (90.0)	23 (92)	18 (86)	6 (86)	7 (100)
Had a partner, n (%)	37 (62.0)	18 (72)	15 (71)	2 (29)	2 (29)
Had ≥1 child, n (%)	9 (15.0)	3 (12)	5 (24)	1 (14)	0 (0)
Current smoker, n (%)	6 (10.0)	4 (16)	2 (10)	0 (0)	0 (0)
Former smoker, n (%)	16 (27.0)	7 (28)	7 (33)	1 (14)	1 (14)

^a^Unpaid advertisements included printed posters and websites.

^b^Traditional media included radio interviews and a published interview.

^c^Referrals included referrals from health care providers and word of mouth.

### Cost of Recruitment

For Facebook advertisements, the research team members spent approximately 15 hours in preparing the advertisements and liaising with Facebook personnel for advertisement approval. The time investment was 0.2 hours per enrolled participant. In terms of cost, each of the 3 advertisements cost Can $300 (US $238.30), adding up to a total of Can $900 (US $714.89), and the time-based compensation per research assistant was Can $375 (US $297.87; 15 hours at Can $25 [US $19.86] per hour). The cost per participant enrolled in the trial from Facebook advertisements was Can $18.21 (US $14.46).

Costs for unpaid advertisements were incurred for the time spent by the research team on developing posters (~10 hours at Can $25 [US $19.86] per hour), distributing them (~150 hours at Can $25 [US $19.86] per hour), and printing them (Can $300 [US $238.30]). The time investment per enrolled participant was 1.6 hours. The cost per participant enrolled in the trial from unpaid advertisements was Can $43.88 (US $34.85).

The trial incurred no direct costs for traditional media and referrals. For recruiting participants from traditional media, a study investigator (JLB) spent 3 hours. The time investment per enrolled participant was 0.2 hours. For referrals, the time investment was approximately 0.1 hours per enrolled participant for 2 hours invested by the study investigators.

## Discussion

### Principal Findings

A combination of 4 strategies facilitated successful participant recruitment in this pilot randomized controlled trial among women of reproductive age living with PCOS. Each of these methods was useful in meeting the participant recruitment target. Unpaid advertisements and Facebook advertisements yielded the greatest number of potential participants, while traditional media was most successful in recruiting participants who completed the trial. The cost per enrolled participant and the time investment by members of the research team was lower for Facebook advertisements than for unpaid advertisements.

The published data on the effectiveness of recruitment strategies for women with PCOS are conflicting. One study examining the effect of diet on weight loss and the risk of endometrial cancer in women with PCOS only recruited 11 of 40 desired participants through multiple recruitment strategies including flyers posted in a specialty clinic, referrals by health care providers, and the inclusion of trial information on a clinic’s website [4]. Furthermore, Pastore and Dalal [6] evaluated the effect of acupuncture in 70 women living with PCOS recruited from among 89 participants as their estimated cohort size, in 36 months. They reported that continual enrollment required multiple recruitment strategies including posters, emails, website listings, direct mail, physician referrals, and traditional advertising in the form of TV, radio, and newspapers. Similarly, we found that multiple concurrent recruitment strategies were necessary to achieve our recruitment target.

Facebook advertisements were used successfully as a recruitment strategy in a previous trial involving women with PCOS [5], which used several recruitment strategies including Facebook advertisements, online advertisements, referrals, and flyers. Of these, Facebook advertisements yielded the highest proportion of potential participants. In our experience, Facebook advertisements were effective in recruiting potential trial participants in 5 weeks of running the advertisements; however, they recruited a lower proportion of potential participants than unpaid advertisements. A recent systematic review [13] examining the role of Facebook advertisements in participant recruitment reported that when compared with traditional recruitment methods including the television, radio, and print media, Facebook advertisements had shorter recruitment periods and lower costs and may have allowed for the inclusion of hard-to-reach demographics including young women.

In our trial, sole dependance on referrals from health care providers would not have been effective in recruiting the number of participants required for this trial. Over 12 months, only 16 potential participants were referred by health care providers. Women living with PCOS often only present for PCOS diagnosis and management if they are concerned by one or more symptoms such as difficulty losing weight, hirsutism, or infertility [14]. They may also prefer immediate treatment for those symptoms and thus may be reluctant to engage in a clinical trial that excludes women taking medical therapy that may affect ovulation. These women may also be motivated to engage in a lifestyle program that involves exercise training as a part of active management of PCOS and might be less likely to enroll in a trial where they may randomly be assigned to a no-exercise control group for 6 months.

### Strengths and Limitations

The notable strength of this study is its provision of detailed data on the comparative success of recruitment strategies used in a pilot randomized controlled trial involving women of reproductive age living with PCOS. There are several limitations to consider when reviewing our results. First, participants who were recruited through word of mouth were not asked specifically how the individual who informed them about the study learned about it. Thus, the reach of our other recruitment strategies (unpaid advertisements, Facebook advertisements, or traditional media) may have been underestimated. Second, we did not use paid traditional media advertisements (ie, television, radio, or newspapers) in our pilot trial, which may be used in a larger trial; therefore, we could not compare the relative success of our recruitment strategies with that of traditional paid media advertisements. Finally, we did not collect data on race, ethnicity, or socioeconomic status to explore the effectiveness of each recruitment strategy on the basis of these variables. Future studies are needed to explore the effect of participant recruitment strategies on participant diversity.

### Conclusion

In conclusion, all 4 recruitment strategies used in this trial were important for recruiting women living with PCOS, which was evident from the finding that none of the participants was recruited through more than 1 strategy. Facebook advertisements and unpaid advertisements yielded the largest number of participants enrolled in the trial. Facebook advertisements were more cost-effective and required less time investment than unpaid advertisements. In future trials, multiple concurrent recruitment strategies should be used to increase participant recruitment.

## Abbreviations

CAET: continuous aerobic exercise training
HIIT: high-intensity interval training
PCOS: polycystic ovary syndrome
